# Comment on “*Pneumocystis jirovecii* Pneumonia in a HIV-Infected Patient with a CD4 Count Greater Than 400 Cells/*μ*L and Atovaquone Prophylaxis”

**DOI:** 10.1155/2023/9793264

**Published:** 2023-10-23

**Authors:** Gilles Nevez, Claire Hoffmann, Solène Le Gal

**Affiliations:** ^1^Université de Brest, Université d'Angers, IRF, Brest, France; ^2^Laboratory of Parasitology and Mycology, Brest University Hospital, Brest, France

We read with interest the article by Sullivan and colleagues [1], who described a case of *Pneumocystis* pneumonia (PCP) occurrence in a HIV-infected patient without severe defect of CD4^+^T-cell count in blood and despite PCP prophylaxis by atovaquone. The HIV infection was correctly controlled by highly active antiretroviral therapy (HAART), but the patient developed PCP after corticosteroid treatment (prednisone, 40 mg/day, 24 days) for hypersensitivity pneumonitis. PCP diagnosis was based on clinical symptoms, chest CT-scan showing bilateral round-glass opacities, and microscopic detection of the fungus in lung biopsies. In addition, at the same time, the value of CD4^+^T-cell count in blood was 487 cells per mm^3^; this is above the usual threshold of 200 cells per mm^3^ below which the risk of PCP is high due to cellular immunodeficiency [2, 3]. However, the diagnosis of overt PCP was clear despite the value of CD4^+^T-cell count being clearly higher than 200 cells per mm^3^. Since the patient presented some markers of immunocompetency and because PCP in these circumstances is highly unusual, the authors reported and discussed their case.

We would like to add some additional observations to the discussion on this case. Beyond the regrettable absence of PCR for *P. jirovecii* detection in the BAL specimen—this technique would have been useful given its high sensitivity—we would like to focus on the following three aspects: First, in addition to the absolute values of CD4^+^ T cells, it would have been interesting to include the values of CD4^+^T-cell percentages as well as the CD4^+^/CD8^+^ ratio. Indeed, although the usefulness of these two parameters is still a subject of debate [4–7], the thresholds of 14%–20% [2, 3, 8–10] and 0.30 [10], respectively, have been reported as additional criteria to evaluate the immunodeficiency of HIV-infected patients and the risk of opportunistic infections. The patient discussed in [1] may have had low CD4^+^ percentages and CD4^+^/CD8^+^ ratios and consequently may have been immunosuppressed and at risk for PCP occurrence, despite the absolute values of CD4^+^ T cells.

Second, cotrimoxazole is the first line of PCP prophylaxis and treatment, while atovaquone represents only a secondary line [11, 12]. In France, atovaquone has been approved for PCP treatment but not for PCP prophylaxis. Despite this restriction in approval, the drug is widely used by clinicians for PCP prophylaxis in patients with contraindications to cotrimoxazole. In this context, we recently reported a newly described mutation (A 144 V) on the gene coding cytochrome b (cyt b), the target of atovaquone, associated with prophylaxis failure among heart transplant recipients (HTR) involved in a PCP outbreak [13]. The mutation conferred diminished sensitivity of *P. jirovecii* to the drug, resulting in the spread of *P. jirovecii* organisms among HTR with long-term atovaquone prophylaxis. Thus, it would be interesting to investigate the cyt b gene of the *P. jirovecii* isolate from Sullivan and colleagues' patient.

Third, we are uncomfortable with a nomenclature misuse, although it could appear anecdotal to the reader. Although the term *Pneumocystis jirovecii* pneumonia (see first line of manuscript summary), in reference to a pneumonia caused by *Pneumocystis jirovecii*, is grammatically correct, it could be considered incorrect nomenclature. The PCP acronym initially referred to “*Pneumocystis carinii *pneumonia.” However, since the early 2000s, the acronym has been redefined so that now PCP stands for *Pneumocystis *pneumonia (see reference [14], page 185, 3^rd^ paragraph, and reference [15]). Indeed, in the course of international workshops on opportunistic protists (IWOP) meetings, a committee decided to keep the PCP acronym, despite the new name *Pneumocystis jirovecii* referring to the human-specific fungus, with *Pneumocystis carinii* referring only to the rat-specific fungus. Be that as it may, the misuse was only noted in the summary and PCP acronym was correctly used by Sullivan and colleagues throughout the remaining manuscript. Moreover, considering the present classification of *Pneumocystis* sp. in the kingdom of fungi, the stages sporozoites and trophozoites must be designated as ascospores and trophic forms, respectively. These stages are visualized by Wright–Giemsa stain, whereas methenamine-silver and toluidine blue O stains render it possible to visualize only the asci, formerly designated as cysts. Conversely, Gram-Weigert stain renders it possible to visualize all *Pneumocystis* stages.

We thank Sullivan and colleagues for their interesting case report.
